# Overexpression of *wbkF* gene in *Brucella abortus* RB51WboA leads to increased O-polysaccharide expression and enhanced vaccine efficacy against *B. abortus* 2308, *B. melitensis* 16M, and B. *suis* 1330 in a murine brucellosis model

**DOI:** 10.1371/journal.pone.0213587

**Published:** 2019-03-11

**Authors:** Neha Dabral, Grant N. Burcham, Neeta Jain-Gupta, Nammalwar Sriranganathan, Ramesh Vemulapalli

**Affiliations:** 1 Department of Comparative Pathobiology, College of Veterinary Medicine, Purdue University, West Lafayette, Indiana, United States of America; 2 Department of Veterinary Pathobiology, College of Veterinary Medicine and Biomedical Sciences, Texas A&M University, College Station, Texas, United States of America; 3 Department of Biomedical Sciences and Pathobiology, VA-MD College of Veterinary Medicine, Virginia Tech, Blacksburg, Virginia, United States of America; Instituto Butantan, BRAZIL

## Abstract

*Brucella abortus* RB51 is an attenuated, stable, spontaneous rough mutant derived in the laboratory from the virulent strain *B*. *abortus* 2308. Previous studies discovered that the *wboA* gene, which encodes a glycosyltransferase required for synthesis of the O-polysaccharide, is disrupted in strain RB51 by an IS*711* element. However, complementation of strain RB51 with a functional *wboA* gene (strain RB51WboA) does not confer it a smooth phenotype but results in low levels of cytoplasmic O-polysaccharide synthesis. In this study, we asked if increasing the potential availability of bactoprenol priming precursors in strain RB51WboA would increase the levels of O-polysaccharide synthesis and enhance the protective efficacy against virulent *Brucella* challenge. To achieve this, we overexpressed the *wbkF* gene, which encodes a putative undecaprenyl-glycosyltransferase involved in bactoprenol priming for O-polysaccharide polymerization, in strain RB51WboA to generate strain RB51WboAKF. In comparison to strain RB51WboA, strain RB51WboAKF expressed higher levels of O-polysaccharide, but was still attenuated and remained phenotypically rough. Mice immunized with strain RB51WboAKF developed increased levels of smooth LPS-specific serum antibodies, primarily of IgG2a and IgG3 isotype. Splenocytes from mice vaccinated with strain RB51WboAKF secreted higher levels of antigen-specific IFN-γ and TNF-α and contained more numbers of antigen-specific IFN-γ secreting CD4^+^ and CD8^+^ T lymphocytes when compared to those of the RB51 or RB51WboA vaccinated groups. Immunization with strain RB51WboAKF conferred enhanced protection against virulent *B*. *abortus* 2308, *B*. *melitensis* 16M and *B*. *suis* 1330 challenge when compared to the currently used vaccine strains. Our results suggest that strain RB51WboAKF has the potential to be a more efficacious vaccine than its parent strain in natural hosts.

## 1. Introduction

Bacteria belonging to the genus *Brucella* are Gram-negative, facultative intracellular coccobacilli that primarily replicate in the monocyte-macrophage lineage of host cells. Some *Brucella* species are the causative agents of brucellosis, a chronic disease that can cause abortion in pregnant animals and undulant fever in humans. Smooth *Brucella* isolates contain an outwardly extending O-polysaccharide (O-PS) as part of their lipopolysaccharide (LPS) structure while isolates exhibiting a rough morphology lack the O-PS [1–5]. O-PS of *Brucella* is a linear homopolymer of 4,6-dideoxy-4-formamido-α-D-mannopyranosyl (perosamine) subunits [6,7]. Smooth LPS protects *Brucella* from complement-mediated lysis and the microbicidal properties of host phagocytic cells [1, 8]. Consequently, smooth *Brucella* strains are more virulent than their rough counterparts, which are typically attenuated [1–3]. The O-PS is also an immunodominant component of LPS; infected animals develop antibodies to it. These anti-O-PS antibodies play a role in enhancing protection against virulent *B*. *abortus*, *B*. *melitensis* and *B*. *suis* in mouse models of infection [9–12]. However, similar to other intracellular pathogens, cell-mediated immunity (CMI) plays a central role in acquired resistance against brucellosis.

*B*. *abortus* strain RB51 is a rough, genetically stable, attenuated, spontaneous mutant of virulent *B*. *abortus* 2308. It is used as a licensed live vaccine for bovine brucellosis in the United States and several other countries [4]. The strain RB51 appears to produce low levels of M-like O-PS, but it does not induce detectable levels of antibodies to smooth LPS in vaccinated hosts [4, 13]. As such, protection conferred by RB51 is mediated through CMI. Previous studies discovered that strain RB51 carries a disruption in *wboA* gene, which encodes a glycosyltransferase required for the synthesis of O-PS [14]. Complementing strain RB51 with a functional *wboA* gene (strain RB51WboA) resulted in the expression of low levels of O-PS. The expressed O-PS remained in the cytoplasmic compartment of strain RB51WboA and the bacteria remained rough in their phenotypic and attenuation characteristics [15]. When compared with strain RB51, vaccination with strain RB51WboA induced a higher level of protection against *B*. *abortus* 2308 and *B*. *melitensis* 16M in mice [15, 16].

The complete biosynthetic pathway for smooth LPS in *Brucella* is not yet fully characterized. However, several genes have been shown to be required for the O-PS and smooth LPS synthesis [17]. These genes include the homologs of *wzm* and *wzt* that encode for the subunits of an ATP-binding cassette (ABC) transporter [17]. Accordingly, the O-PS in *Brucella* is postulated to be synthesized by the ABC transporter-dependent pathway [18]. The O-PS is first assembled by addition of monomers onto bactoprenol linked-oligosaccharide intermediate and the completed unit is then translocated across the inner membrane. Comparative nucleotide sequence analysis of the genes that are essential for O-PS biosynthesis did not reveal any other apparent mutation, other than in *wboA*, that could affect the smooth LPS synthesis in strain RB51 [19].

The objective of this study was to determine if increasing the potential availability of bactoprenol primed-oligosaccharide precursors in strain RB51WboA would increase the levels of O-PS and enhance the protective efficacy against virulent *Brucella* challenge. For this, we overexpressed *wbkD* or *wbkF*, which encode enzymes that catalyze steps leading to the synthesis of bactoprenol primers for O-PS polymerization [17], in strain RB51WboA. Our results show that *wbkF*, but not *wbkD*, overexpression in strain RB51WboA leads to increased O-PS expression. Also, *wbkF* overexpression in strain RB51WboA enhances its protective efficacy against challenge with virulent *B*. *abortus* 2308, *B*. *melitensis* 16M and *B*. *suis* 1330.

## 2. Materials and methods

### 2.1. Ethics statement

The protocols of the mice experiments performed in this study were approved by the Institutional Animal Care and Use Committees at Purdue University (Approval # 1112000488) and Virginia Tech (Approval # CVM-10-048 and 15-072CVM). The animal studies were conducted in strict accordance with the recommendations in the Guide for the Care and Use of Laboratory Animals of the National Institutes of Health [20]. Blood was collected from mice under anesthesia by puncture of the retro-orbital plexus. For anesthesia, regulated concentration of anesthetic mixture (oxygen and isoflurane) was administered via a commercially available rodent anesthesia machine (Vetamac, Inc., Rossville, Indiana). Following blood collection, a drop of proparacaine hydrochloride ophthalmic solution (Bausch & Lomb, Tampa, Florida) was placed on the eye to reduce pain. Mice infected with virulent *B*. *abortus* 2308, *B*. *melitensis* 16M or *B*. *suis* 1330 do not develop clinical disease or exhibit any signs of suffering for the duration of the experiments conducted in this study. Therefore, no humane endpoints were utilized for the mice in this study.

### 2.2. Bacterial strains

*B*. *abortus* strains RB51, RB51WboA, and 2308, *B*. *melitensis* strain 16M, *B*. *suis* strain 1330 and *B*. *neotomae* were from our culture collection. The plasmid constructs were prepared using *Escherichia coli* strain DH5α (Invitrogen, Carlsbad, CA). Tryptic soy broth (TSB) and tryptic soy agar (TSA) were used as a growth media for all bacterial strains. The growth media was supplemented with ampicillin at 100 μg/ml or chloramphenicol at 30 μg/ml for selection of the bacteria harboring plasmids. All protection experiments with virulent *Brucella* were performed in a BSL-3 facility approved for select agents work. *B*. *neotomae* was used as positive control of smooth LPS phenotype in some experiments.

### 2.3. Generation of recombinant strains RB51WboAKF and RB51WboAKD

Genes *wbkF* and *wbkD*, along with the upstream flanking regions containing their putative promoters, were amplified using custom-designed primer-pairs (Table 1) by PCR. *B*. *abortus* 2308 genomic DNA was used as template. Forward and reverse primers containing *Kpn*I and *Xho*I restriction sites, respectively, were used to amplify *wbkD* and *wbkF*. The amplified fragments were cloned in pGEM-T Easy plasmid (Promega, Madison, WI). Sequence analysis was used to confirm the integrity of the cloned nucleotide sequences. The inserts were subsequently excised from the pGEM-T plasmids using the restriction enzymes specific to the respective restriction sites engineered into the primers. The fragment containing the *wbkF* gene was cloned into the same sites of pAB3, a pBBR1MCS-based plasmid containing the *wboA* gene fragment [3]. The fragment containing the *wbkD* gene was similarly cloned into pAB3. The resulting plasmids, pBB1/wboAKF (containing *wboA* and *wbkF*) and pBB1/wboAKD (containing *wboA* and *wbkD*) were electroporated into strain RB51 as per previously described procedure [21] to generate strains RB51WboAKF and RB51WboAKD, respectively.

**Table 1:** Primers used in this study for PCR gene amplification and for RT-PCR.

PCR		
Gene	Forward Primer (5’-3’)	Reverse Primer (5’-3’)
*wbkD*	TTTT*GGTACC*GGGCGTATGGTTGCGG	TTTT*CTCGAG*CGCTTCAGGAAGCTATGACC
*wbkF*	TTTT*GGTACC*GAGCTTTGACATTATCCGTG	TTTT*CTCGAGG*TCATAGCTTCCTGAAG
RT-PCR Analysis		
Gene	Forward Primer (5’-3’)	Reverse Primer (5’-3’)
*wbkD*	ATGATCCTGCTCGCGGGAACT	GCGAGCGAAGGATCGTAGAA
*wbkF*	ATGAGTGTGGCAACGGCTGC	AGTGGTGGCCCATGTGGC
*If-1*	ATGGCGAAAGAAGTCCT	ACTAGAACCTTGTCACCGGC

### 2.4. SDS-PAGE and Western blotting

SDS-PAGE and Western blot analyses were carried out as previously described [22]. Briefly, antigen extracts of RB51, RB51WboA, RB51WboAKF, RB51WboAKD and *B*. *neotomae* were separated on a 12.5% denaturing polyacrylamide gel by electrophoresis. For Western blotting, the separated antigens were transferred onto a nitrocellulose membrane. The membrane was blocked in 5% skim milk for 3 h and was subsequently reacted with appropriately diluted *Brucella* O-PS specific rat monoclonal antibody, Bru-38 [4]. Following reaction with a horseradish peroxidase labelled-secondary antibody (KPL, Gaithersburg, MD), a colorimetric substrate (TMB substrate, KPL, Gaithersburg, MD) was used to detect the binding of the primary antibody. The separated antigens on the polyacrylamide gel were also stained with Coomassie Brilliant Blue.

### 2.5. RNA isolation and reverse transcription (RT)-PCR

Cultures of RB51, RB51WboA, RB51WboAKF, RB51WboAKD, and *B*. *neotomae* were grown in TSB at 37°C. Total RNA was isolated from the bacteria using the miRCURY RNA isolation kit (Exiqon Inc., Woburn, MA) according to the manufacturer’s protocol. Contaminating DNA was removed using Turbo DNA-free kit (Life Technologies, Grand Island, NY) following manufacturer's instructions. RNA purity and concentration were evaluated using electrophoresis and by spectrophotometric analysis (Nanodrop ND-1000, Nanodrop, Wilmington, DE).

The expression level of *wbkD* and *wbkF* mRNA in different strains was determined using a reverse transcription-polymerase chain reaction assay. The mRNA levels of the house keeping gene, translation initiation factor IF-1, were also determined and used for normalization of the *wbkD* and *wbkF* transcript levels [23]. Briefly, using 100 ng of the extracted total RNA, target mRNA sequences were amplified using Superscript III Platinum SYBR green one-step qRT-PCR kit (Life Technologies, Grand Island, NY). Gene specific primers were used for the RT-PCR assay (Table 1). The RT-PCR assay was performed in triplicates for each sample using Stratagene MX3000P thermocycler (Stratagene, La Jolla, CA). The samples were incubated at 50°C for 15 min for cDNA synthesis. Following another incubation step at 95°C for 5 min, the samples were subjected to 40 cycles (30 sec at 95°C, 1 min at 55°C, 30 sec at 72°C). The fold change was calculated using the comparative threshold method [24].

### 2.6. Polymyxin B sensitivity assay

Polymyxin B (PmB) sensitivity assay was performed as described previously [1, 25]. Briefly, strains RB51 and RB51WboAKF, and *B*. *neotomae* were grown to log phase in broth cultures. The bacterial cultures were pelleted by centrifugation at 4,200×g, resuspended in sterile 10 mM phosphate buffer (pH 7.2) at approximately 1×10^4^ CFU/ml, and incubated for 1 h with different concentrations of Polymyxin B (Sigma-Aldrich, St. Louis, MO) in the range of 0–50 μg/ml. Immediately following the incubation, the bacterial cell suspensions were diluted 1:10 and 1:100 in sterile 10 mM phosphate buffer and 50 μl was plated on TSA plates. Average results of three independent assays were expressed as percentage of bacteria surviving in the absence of Polymyxin B.

### 2.7. Immuno-electron microscopy

Freshly grown cultures of strains RB51 and RB51WboAKF and *B*. *neotomae* were used. The bacterial cells were fixed in 4% paraformaldehyde and 0.1% glutaraldehyde in 0.1 M sodium phosphate buffer. Subsequently, they were pelleted in 2% agarose (Sigma-Aldrich), dehydrated with a graded ethanol series to 85% ethanol and infiltrated with LR White resin. Polymerization was done overnight at 53°C in flat embedding molds (Ted Pella, Inc.). Sections were cut on a Reichert-Jung Ultracut E ultramicrotome and collected on 100 mesh formvar-coated nickel grids. Grids were incubated on 50 mM glycine for 30 min and then on blocking solution (0.1 M sodium phosphate buffer containing 0.5% Aurion BSA-c, 1% normal goat serum, and 0.2% Tween 20) for 1 h. Rabbit anti-smooth LPS serum (purified *B*. *neotomae* LPS was used as the immunogen to raise antibodies in rabbits at a commercial source) was used as primary antibody. Rabbit antibodies were used because of the unavailability of sufficient quantity of Bru-38 monoclonal antibody. Primary antibody incubation was done overnight at 4°C with a 1:100 dilution of the anti-smooth LPS antibody in blocking solution. Grids were rinsed seven times and incubated on goat anti-rabbit secondary antibody conjugated with 10 nm gold in blocking solution for 90 min. Grids were washed six times and stained with 2% uranyl acetate. Images were acquired on a FEI Tecnai G^2^ 20 electron microscope equipped with a LaB_6_ source and operating at 100 kV.

### 2.8. Mice experiments

Female BALB/c mice of 4 to 6 weeks of age were used for all the studies. Mice were purchased from a commercial source (Harlan Laboratories, USA), and housed in cages with microisolator tops at up to 4 mice per cage. Feed and water were provided ad libitum. Housing conditions included standard 12 h light/dark cycle, controlled humidity (55%) and room temperature (22°C). Mice were allowed to acclimatize to the new environment for 1 week.

To determine the persistence of the recombinant strain, groups of 12 mice each were immunized by intra-peritoneal (i.p.) inoculation with 2×10^8^ CFU of strain RB51 or RB51WboAKF. Three mice from each group were euthanized by CO_2_ asphyxiation, followed by cervical dislocation on days 1, 7, 21 and 28 post-immunization (p.i.), and spleens, livers and lungs were aseptically collected. The bacterial CFUs in the mouse organs were enumerated as previously described [4]. Briefly, each spleen, liver and lung was homogenized with a tissue mincer and serially diluted 10-fold in TSB. Fifty μl from each serial dilution was drop plated, incubated at 37°C for up to 5 days, and the number of bacterial CFUs were enumerated.

To determine systemic cytokine response to vaccination, groups of 15 mice were each inoculated i.p. with 2×10^8^ CFU of RB51 or RB51WboAKF. A group of mice inoculated with saline was used as a control. Blood was collected from 5 mice of each group at days 1, 7 and 28 p.i. The serum was separated from the clotted blood and concentrations of the cytokines IL-2, GM-CSF, IFN-γ, TNF-α, IL-4, IL-5, IL-10 and IL-12p70 were determined using Bio-Plex Pro Mouse Cytokine Th1/Th2 Assay (Bio-Rad, Hercules, CA) according to the manufacturer’s instructions.

To assess the serological responses, groups of four BALB/c mice were each immunized by i.p. inoculation with 2×10^8^ CFU of RB51, RB51WboA or RB51WboAKF. A group inoculated with saline alone was used as a control. Blood was collected from mice at 3 and 6 weeks p.i. Serum was separated and stored at -20°C until further use for indirect ELISA (see below section 2.9). Also, at 6 weeks p.i., the mice were euthanized by CO_2_ asphyxiation followed by cervical dislocation and their spleens were aseptically collected. The splenocytes were harvested from the spleens and used for the analysis of cell-mediated immune responses (see below section 2.10).

### 2.9. Indirect ELISA

Levels of RB51-specific and smooth LPS-specific serum immunoglobulin M (IgM), IgG, as well as IgG1, IgG2a, IgG2b and IgG3 isotypes were determined using indirect ELISA, as described previously [26]. Briefly, heat-killed RB51 and smooth LPS extracted from *B*. *neotomae* were diluted in carbonate buffer (pH 9.6) and used to coat polystyrene plates (Nunc-Immunoplate with maxisorp surface). Following overnight incubation, the plates were incubated with blocking buffer (5% skim milk in TBS) for 1 h and diluted mouse sera (1:200 in blocking buffer) were added to the wells (50 μl/well). Following washing, appropriately diluted horseradish peroxidase labelled isotype specific conjugates (Southern Biotechnology Associates Inc, Birmingham, Alabama) were added to the wells (50 μl/well). The plates were washed and substrate solution (TMB Microwell peroxidase substrate; KPL, Gaithersburg, MD) was added to each well (100 μl/well). The enzyme reaction was stopped by100 μl of stop solution (0.185 M sulfuric acid) and the absorbance was recorded by a microplate reader (Molecular devices, Sunnyvale, CA).

### 2.10. Culture of splenocytes

Single cell suspension of the splenocytes was prepared as previously described [22]. The erythrocytes were lysed using ACK lysis buffer (Thermo Fisher Scientific, Grand Island, NY) and the splenocytes were cultured in triplicates in 96-well flat-bottomed culture plates (Sigma-Aldrich, St. Louis, MO), 5×10^5^ cells/well for the quantification of cytokines secreted into the culture supernatant and 1×10^6^ cells/well for the flow cytometric analysis of antigen-specific IFN-γ secreting CD4^+^ and CD8^+^ T cells. The splenocytes were cultured in the presence of different stimulants: 10^7^ CFU-equivalents of heat-killed RB51 and RB51WboAKF. Cells stimulated with 2.5 μg/ml of concanavalin A (ConA) and plain media were used as controls.

### 2.11. Quantification of cytokines in the culture supernatants of splenocytes

The splenocytes were cultured with different stimulants and controls (as described above in culture of splenocytes) for 5 days in a humidified incubator with 5% CO_2_ at 37°C. Subsequently, the supernatants were collected and the concentrations of the cytokines were determined as previously described (Materials and Methods 2.8).

### 2.12. Flow cytometric analysis of antigen-specific IFN-γ secreting CD4^+^ and CD8^+^ T cells

A previously described procedure was followed for intracellular IFN-γ staining [27]. The splenocytes were cultured with different stimulants and controls (as described above in culture of splenocytes) for 10 h in a humidified incubator with 5% CO_2_ at 37°C. Brefelin A (Golgistop; Pharmingen) was added to the wells and the plates were incubated for another 6 h. Cells from each treatment were suspended in FACS buffer (PBS containing 1% BSA and 0.2% sodium azide). They were incubated for 30 min on ice with appropriately diluted FITC-conjugated CD8 antibody (BD Pharmigen, clone 53–6.7) and APC-conjugated CD4 antibody (BD Pharmigen, clone L3T4-RM 4.5). Following three washes with FACS buffer, the cells were stained for intracellular IFN-γ with PE-conjugated rat anti-mouse IFN-γ antibody using the cytofix/cytoperm (Pharmingen) according to the manufacturer’s instructions. As an isotype control, cells stained with PE-conjugated rat IgG1 antibody were used. The cells were acquired on BD FACS Canto II Flow cytometer (BD Biosciences, CA, USA). The data were analyzed using BD FACSDIVA version 6 software (BD Biosciences, CA, USA) and the proportion of CD4^+^ and CD8^+^ T cells that secreted IFN-γ was determined.

### 2.13. Mice experiments—Protection studies

Three separate protection experiments were performed. In each experiment, groups of 5 mice each were inoculated by i.p. route with an experimental or positive control reference vaccine (RB51 or S19 or Rev1 or VTRS1). Mice inoculated by the same route with saline solution served as negative controls. Six weeks post-vaccination, mice in each group were challenged by i.p. route with 5×10^4^ CFU of *B*. *abortus* 2308, or *B*. *melitensis* 16M, or *B*. *suis* 1330. Two weeks post-challenge, the mice were euthanized and the bacterial burden in their spleens was enumerated as previously described [4]. In the first experiment, protective efficacy of RB51WboA or RB51WboAKF vaccines at a dose of 2×10^8^ CFU/mouse was tested against challenge with *B*. *abortus* 2308 or *B*. *melitensis* 16M. A group of mice inoculated with RB51 was used as a positive control reference of vaccine efficacy. In the second experiment, protective efficacy of two different doses of RB51WboAKF (1×10^8^ CFU/mouse or 1×10^6^ CFU/mouse) was compared with that of currently used vaccines *B*. *abortus* S19 (1×10^6^ CFU/mouse) and *B*. *melitensis* Rev 1 (1×10^6^ CFU/mouse) against challenge with *B*. *abortus* 2308 or *B*. *melitensis* 16M. In the third experiment, protective efficacy of two different doses of RB51WboAKF (1×10^8^ CFU/mouse or 1×10^6^ CFU/mouse) was compared with that of *B*. *suis* VTRS1 (1×10^6^ CFU/mouse) control against challenge with *B*. *suis* 1330. Protection was determined in log units by subtracting the mean log CFU/spleen of the vaccinated groups from the mean log CFU/spleen of the saline inoculated group.

### 2.14. Statistical analysis

Statistical analysis was performed using GraphPad Prism software (GraphPad, San Diego, CA). ELISA absorbance values, cytokine concentrations and flow cytometry data were analyzed for differences among groups by performing one-way analysis of variance (ANOVA) with post-hoc Bonferroni test. Persistence and protection study data were analyzed using one-tailed t-test modified for unequal variances between different groups.

## 3. Results

### 3.1. O-Polysaccharide expression in strain RB51WboA overexpressing *wbkF* gene

**Fig 1:**
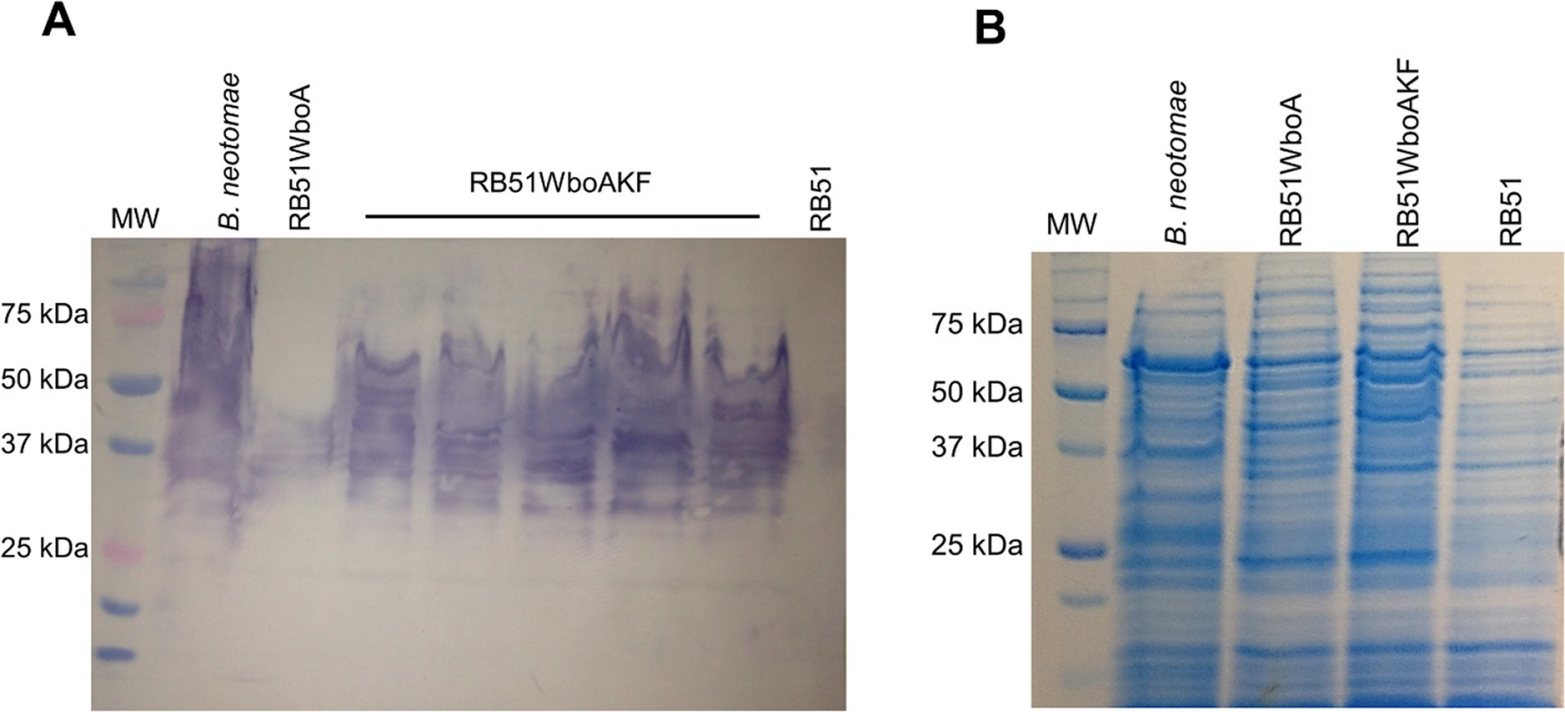
Overexpression of *wbkF* in strain RB51WboA results in O-polysaccharide expression. Whole bacterial antigens were separated by SDS-PAGE and analyzed by (A) Western blotting with O-polysaccharide specific monoclonal antibody, Bru-38, and (B) Coomassie Brilliant Blue staining of the polyacrylamide gel to detect any differences in the expressed protein profiles.

Five separate colonies of strain RB51WboAKF were selected for determination of O-PS expression. Western blot analysis with Bru-38 showed that all five RB51WboAKF colonies expressed O-PS of high molecular weight (Fig 1A). The reactivity profile of RB51WboAKF recombinants was similar to that of *B*. *neotomae*, which served as positive control. In contrast, Bru-38 reacted with a few bands in the 40 kDa range in strain RB51WboA, similar to previous report [15] (Fig 1A). As expected, RB51 did not exhibit any reactivity with Bru-38 [4] (Fig 1A). One of the RB51WboAKF recombinants was selected for all further studies. Strain RB51WboAKD had a similar reactivity pattern as that of strain RB51WboA.

Both strains RB51WboAKF and RB51WboAKD auto-agglutinated in presence of 1% acriflavin.

SDS-PAGE followed by Coomassie Brilliant Blue staining was used to visualize any differences in the protein profiles of the strains RB51 and RB51WboAKF. There was no apparent qualitative difference in the protein profiles of the two strains (Fig 1B).

**Fig 2:**
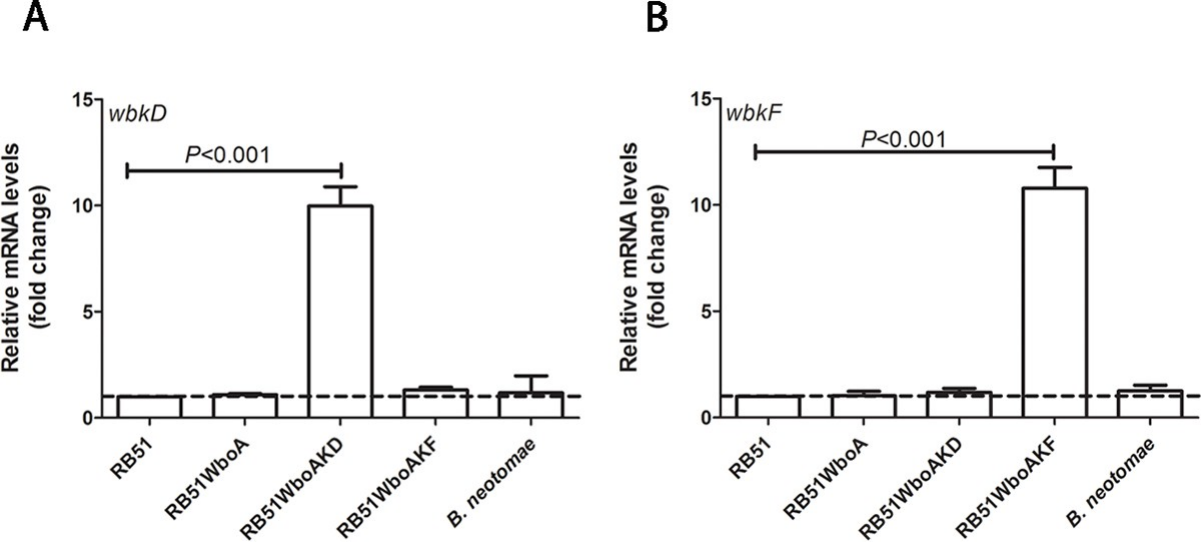
Transcript levels of *wbkF* in RB51WboAKF were nearly ten-fold higher than levels in RB51. RT-PCR detection of (A) *wbkD* and (B) *wbkF* mRNA in strains RB51, RB51WboA, RB51WboAKD, RB51WboAKF and *B*. *neotomae*. Graphs show the mean values of fold change for *wbkD* and *wbkF* transcript levels in the strains RB51WboA, RB51WboAKD, RB51WboAKF and *B*. *neotomae* relative to those of RB51, which were converted to 1. All the values are relative to those of internal control gene IF-1. Results are shown as mean ± standard deviation (n = 3).

The levels of *wbkD* and *wbkF* mRNA transcripts in strains RB51WboAKD and RB51WboAKF were quantified by RT-PCR. Relative to RB51, RB51WboA and *B*. *neotomae*, the mRNA levels of *wbkD* and *wbkF* in strains RB51WboAKD and RB51WboAKF, respectively, were detected to be nearly ten-fold higher (Fig 2A and 2B).

**Fig 3:**
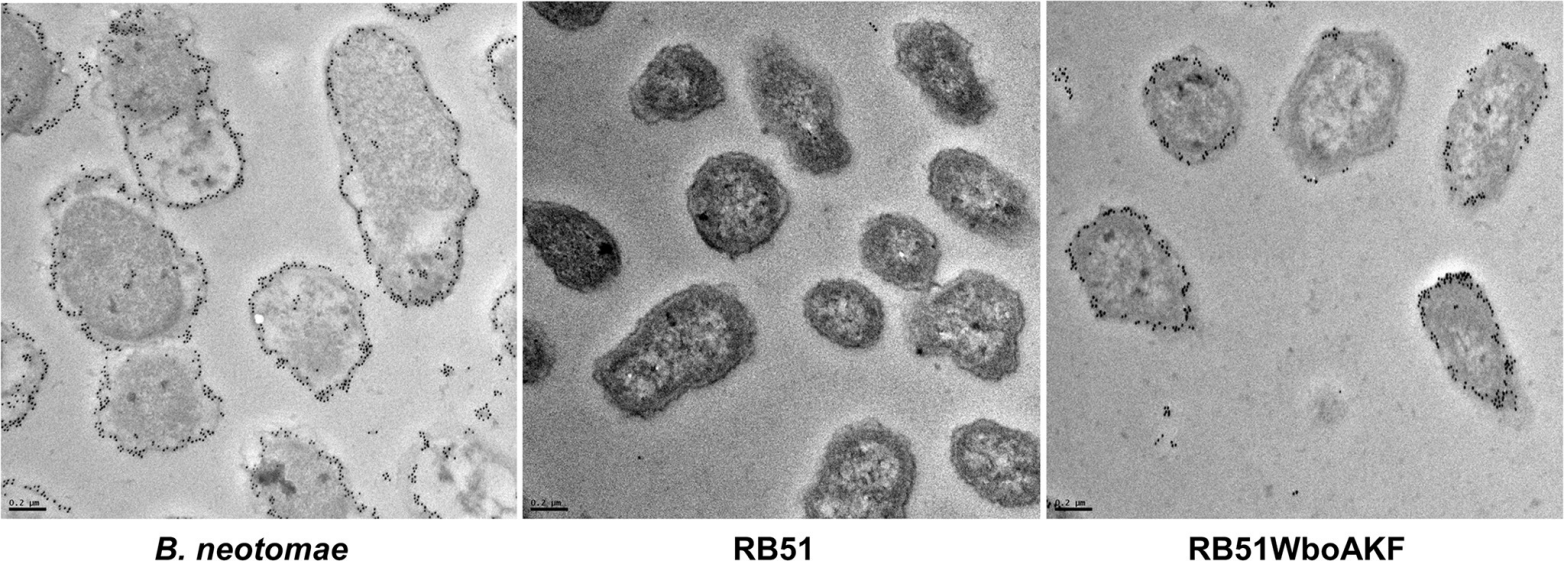
Presence of O-polysaccharide in the cell membrane of strain RB51WboAKF. The sections of *B*. *neotomae*, RB51 and RB51WboAKF were reacted with anti-*B*. *neotomae* LPS followed by gold-labeled goat anti-rabbit IgG. Bars = 0.2 μm.

The location of the expressed O-PS in bacterial cells was determined by immune-electron microscopy. Microscopy of thin sections of RB51WboAKF revealed the presence of O-PS mostly in the outer membrane and on the bacterial cell surface (Fig 3). As expected, no O-PS was detected in strain RB51, while the presence of O-PS in strain *B*. *neotomae* was also mainly detected in the outer membrane and on the cell surface (Fig 3).

**Fig 4:**
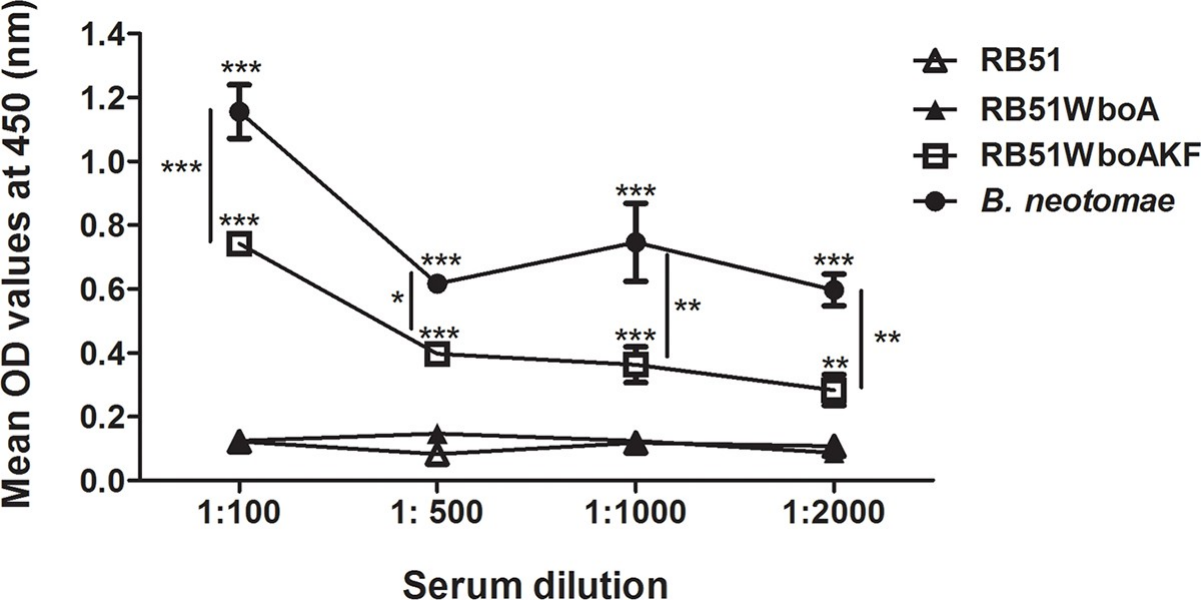
ELISA detection of O-polysaccharide expression on the cell surface of strain RB51WboAKF. RB51, RB51WboA, RB51WboAKF and *B*. *neotomae* whole cells (10^8^ CFU/well) were reacted with specific dilutions of anti-*B*. *neotomae* LPS antibody. The results are shown as mean ± standard deviation (n = 2) of the optical density (OD) at 450 nm of the color developed. Asterisks indicate statistically significant differences from strain RB51. *, *P* < 0.05; **, *P* < 0.01; ***, *P* < 0.001. OD, optical density.

Indirect ELISA with whole bacterial cells as antigen was performed to determine the presence of O-PS expression on the surface of bacterial cells. As expected, RB51 and RB51WboA whole cells did not react with smooth LPS-specific antibody (Fig 4). In contrast, both strain RB51WboAKF and *B*. *neotomae* exhibited significantly higher level of reactivity with the anti-smooth LPS antibody; however, the level of reactivity of *B*. *neotomae* with the antibody was significantly higher (Fig 4).

### 3.2. Sensitivity to Polymyxin B (PmB) antibacterial activity

**Fig 5:**
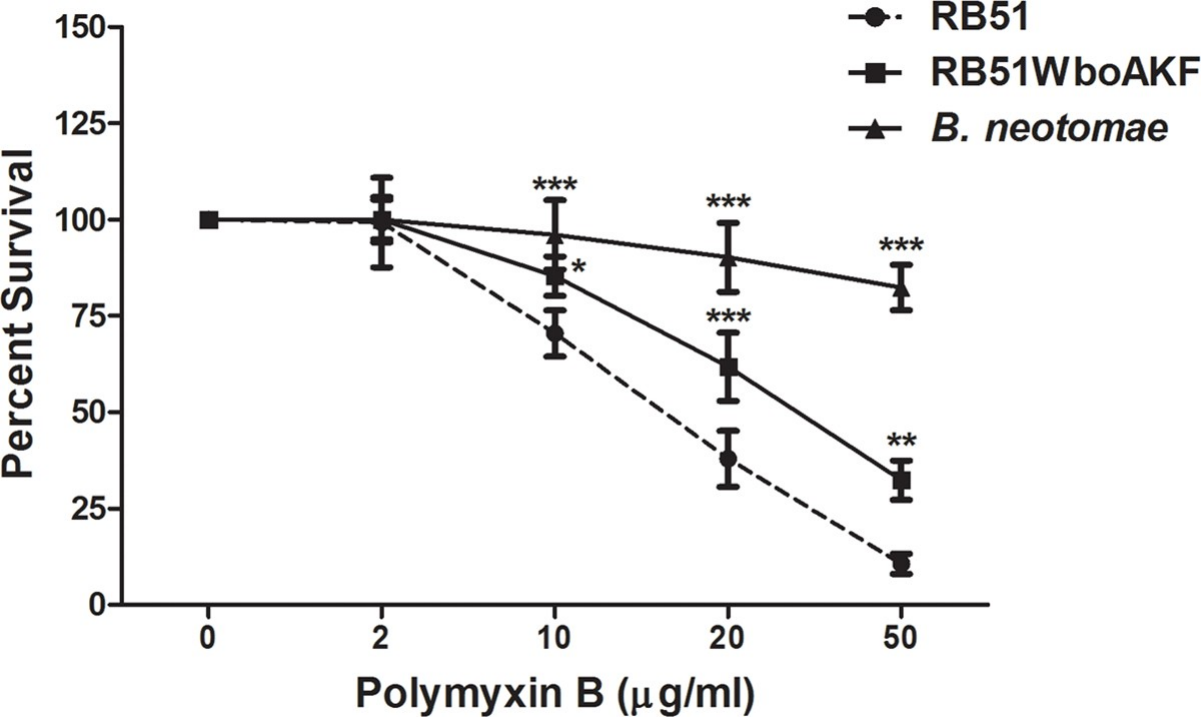
Bactericidal effect of Polymyxin B on strains RB51, RB51WboAKF and *B*. *neotomae*. Average results from three assays are expressed as percentage of the bacteria surviving in the absence of Polymyxin B. Asterisks indicate statistically significant differences from the RB51 strain. *, *P* < 0.05; **, *P* < 0.01; ***, *P* < 0.001.

Strain RB51WboAKF was less sensitive than RB51 to PmB-mediated antibacterial effect at concentrations ≥ 10 μg/ml (Fig 5). The difference was more apparent at higher concentrations (20–50 μg/ml) of PmB (Fig 5). As expected, tested concentrations of PmB had the least effect on strain *B*. *neotomae* which exhibited higher survival percentage when compared to strains RB51 and RB51WboAKF (Fig 5).

### 3.3. Persistence of strain RB51WboAKF in mice

**Fig 6:**
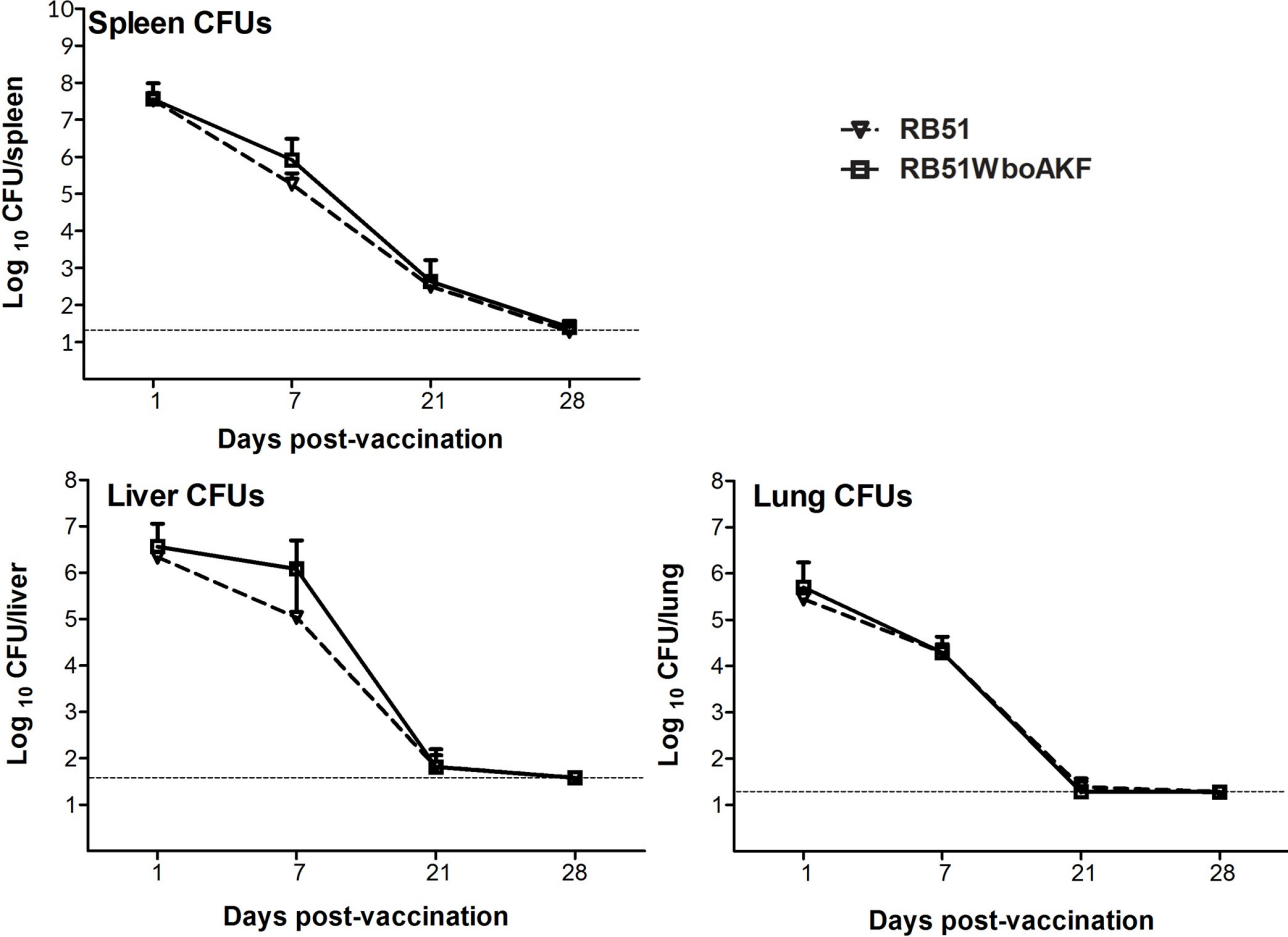
Persistence of strain RB51WboAKF in mice. Mice spleens, livers and lungs were collected at days 1, 7, 21 and 28 after vaccination with live RB51 and live RB51WboAKF. The *Brucella* CFUs in these organs were determined as described in Materials and Methods. Results are shown as mean ± standard deviation (n = 3) of the log CFU of *Brucella* recovered from each organ. Horizontal broken line above the x-axis indicates the lower limit of detection (<20 CFU/spleen and lungs, <40 CFU/liver).

Persistence of strains RB51 and RB51WboAKF in mice organs was compared in order to assess the attenuation characteristics of the strain RB51WboAKF. There was no difference in the persistence pattern of RB51 and RB51WboAKF in spleens, livers and lungs of vaccinated mice at days 1, 7, 21 and 28 p.i. (Fig 6). Both the strains were not detected in the livers and lungs and were present at very low levels in spleens of mice at day 28 p.i. (Fig 6).

### 3.4. Cytokine response of mice to vaccination

**Fig 7:**
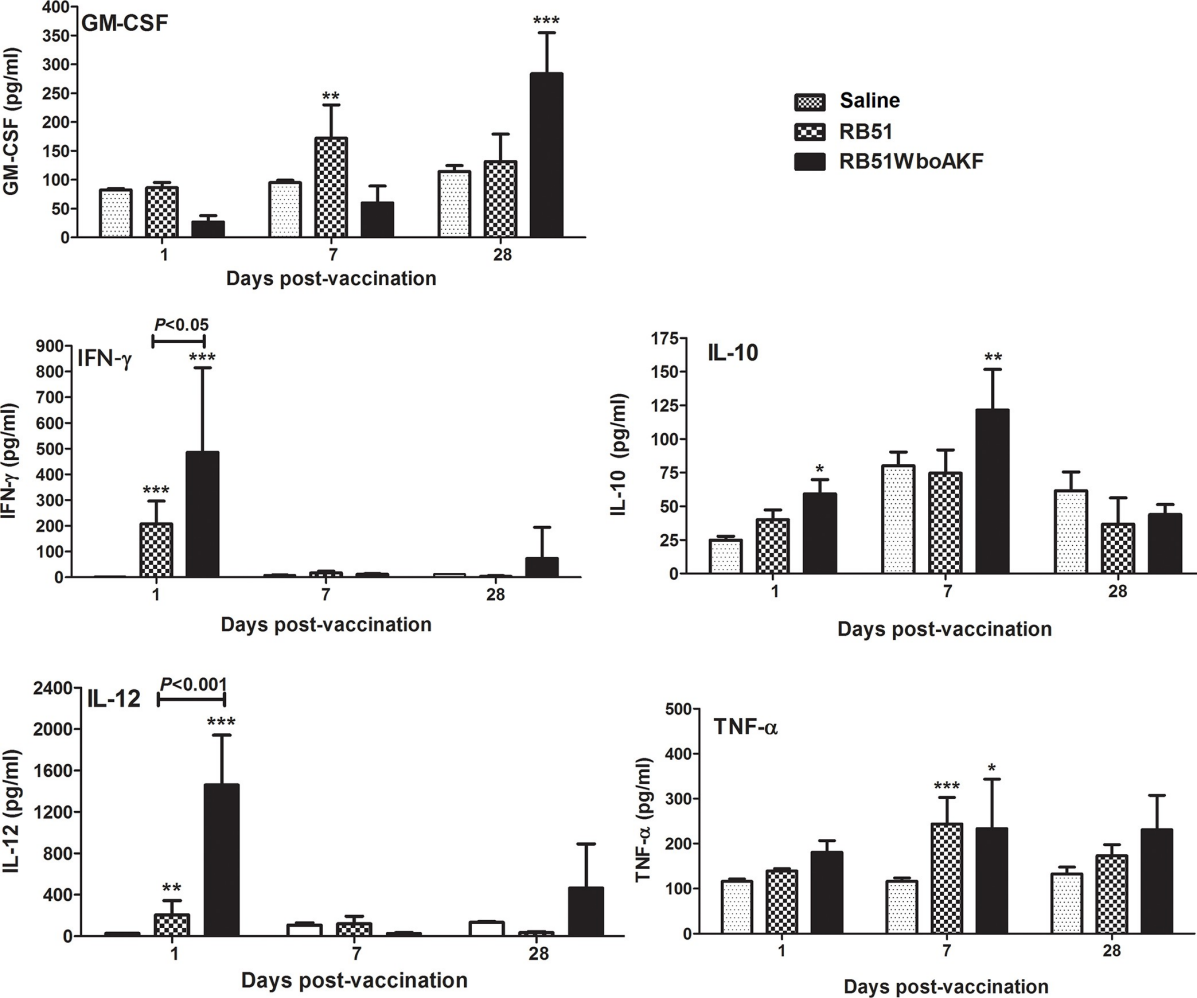
Detection of specific cytokines in the serum of RB51WboAKF vaccinated mice. Mice were immunized intra-peritoneally with 10^8^ CFU-equivalent of live RB51, RB51WboAKF, or inoculated with saline. Values are shown as mean ± standard deviation (n = 5). Asterisks indicate statistically significant differences from the corresponding saline group. *, *P* < 0.05; **, *P* < 0.01; ***, *P* < 0.001.

Systemic cytokine response of mice to vaccination was determined by quantifying the levels of different cytokines in serum. Significantly higher levels of IL-12 and IFN-γ at day 1 and TNF-α at day 7 p.i. were detected in the serum of mice vaccinated with RB51 and RB51WboAKF when compared with the saline-inoculated mice (Fig 7). The levels of IL-12 and IFN-γ were significantly higher at day 1 p.i. in the serum of RB51WboAKF immunized mice than those immunized with RB51 (Fig 7). Moreover, significantly increased levels of GM-CSF at day 28 p.i. and IL-10 at days 1 and 7 p.i. were detected in the serum of mice vaccinated with RB51WboAKF when compared with RB51 vaccinated and saline inoculated mice groups (Fig 7). The concentrations of IL-4 and IL-5 were below the limit of detection in mice of all the groups.

### 3.5. Antigen-specific antibody responses in vaccinated mice

**Fig 8:**
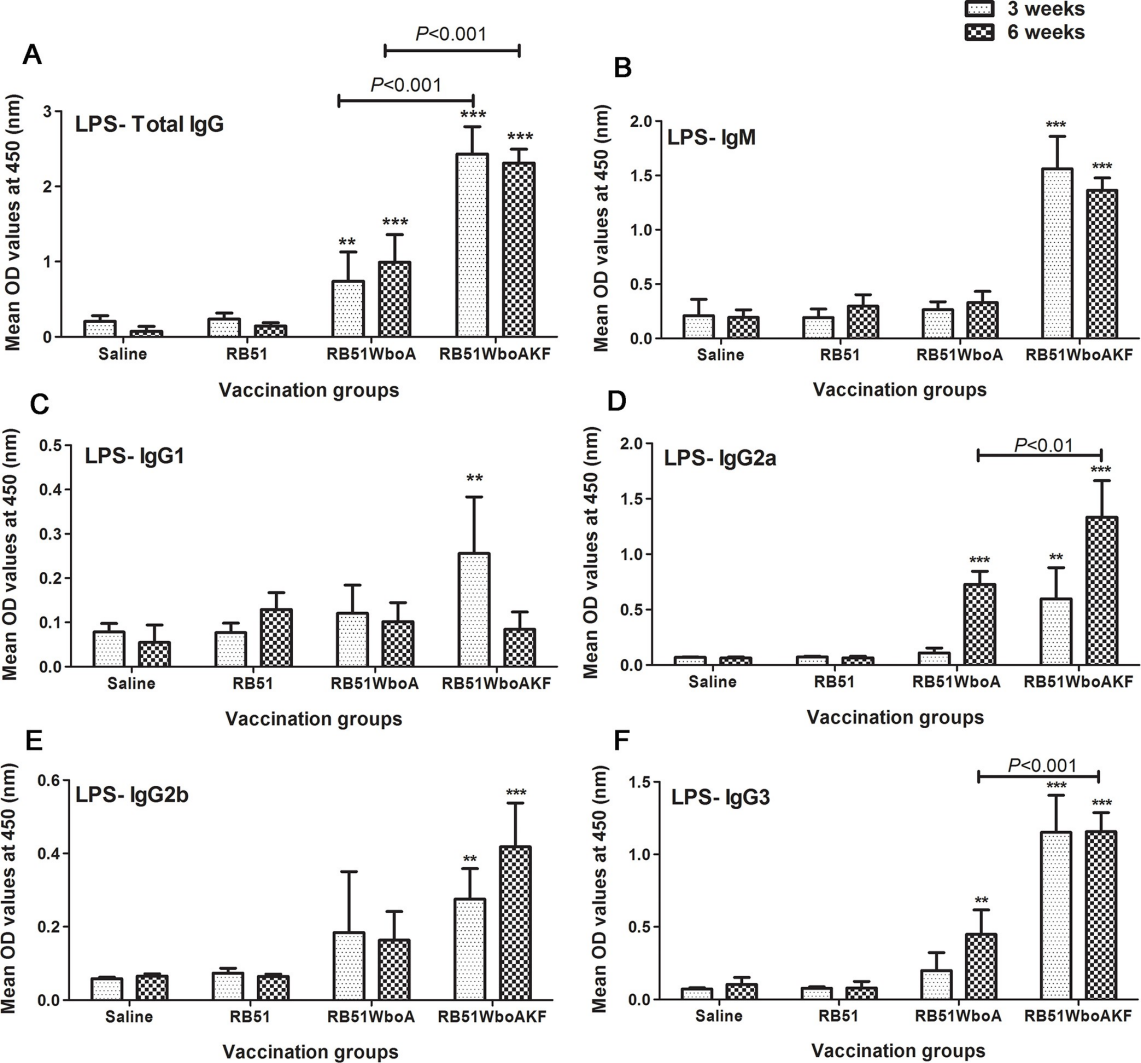
Detection of smooth LPS-specific IgM, IgG, IgG1, IgG2a, IgG2b and IgG3 antibodies in serum of mice inoculated with RB51, RB51WboA, RB51WboAKF or saline. Serum samples were collected at 3 and 6 weeks after vaccination, were diluted 1 in 200 and assayed for the presence of smooth LPS-specific antibodies by indirect ELISA. Results are shown as mean ± standard deviation (n = 4) of absorbance of the color developed. Asterisks indicate statistically significant differences from the corresponding saline group. *, *P* < 0.05; **, *P* < 0.01; ***, *P* < 0.001. OD, optical density.

Higher levels of smooth LPS-specific total IgG antibodies were detected in the serum of mice vaccinated with RB51WboA and RB51WboAKF when compared with RB51 vaccinated and saline-inoculated mice (Fig 8A). Smooth LPS-specific IgG levels were significantly higher in RB51WboAKF vaccinated mice when compared with those vaccinated with RB51WboA (Fig 8A). Mice vaccinated with RB51WboAKF also had significantly higher levels of smooth LPS-specific IgM, IgG2a, IgG2b and IgG3 at 3 and 6 weeks p.i. and IgG1 at 3 weeks p.i. when compared with RB51 vaccinated and saline-inoculated mice group (Fig 8B–8F). In contrast, significantly higher levels of smooth LPS-specific IgG2a and IgG3 antibodies were detected in mice vaccinated with RB51WboA only at 6 weeks p.i. when compared with the RB51 vaccinated and unvaccinated group of mice (Fig 8D and 8F). The levels of smooth LPS-specific IgG2a and IgG3 were significantly higher in the serum of RB51WboAKF vaccinated mice than those vaccinated with RB51WboA (Fig 8D and 8F).

All groups of vaccinated mice (RB51, RB51WboA and RB51WboAKF) had similar levels of antibodies specific to RB51 protein antigens (S1 Fig). Only the level of RB51-specific IgG2a was significantly higher in the serum of RB51WboAKF vaccinated mice when compared to the other vaccinated groups (S1 Fig).

### 3.6. Induction of specific cell-mediated responses in mice

Specific cell-mediated responses were analyzed at 6 weeks p.i. in the vaccinated mice by determining the number of IFN-γ secreting CD4^+^ and CD8^+^ T cells as well as by quantifying cytokines secreted by splenocytes upon *in vitro* stimulation with specific antigens.

**Fig 9:**
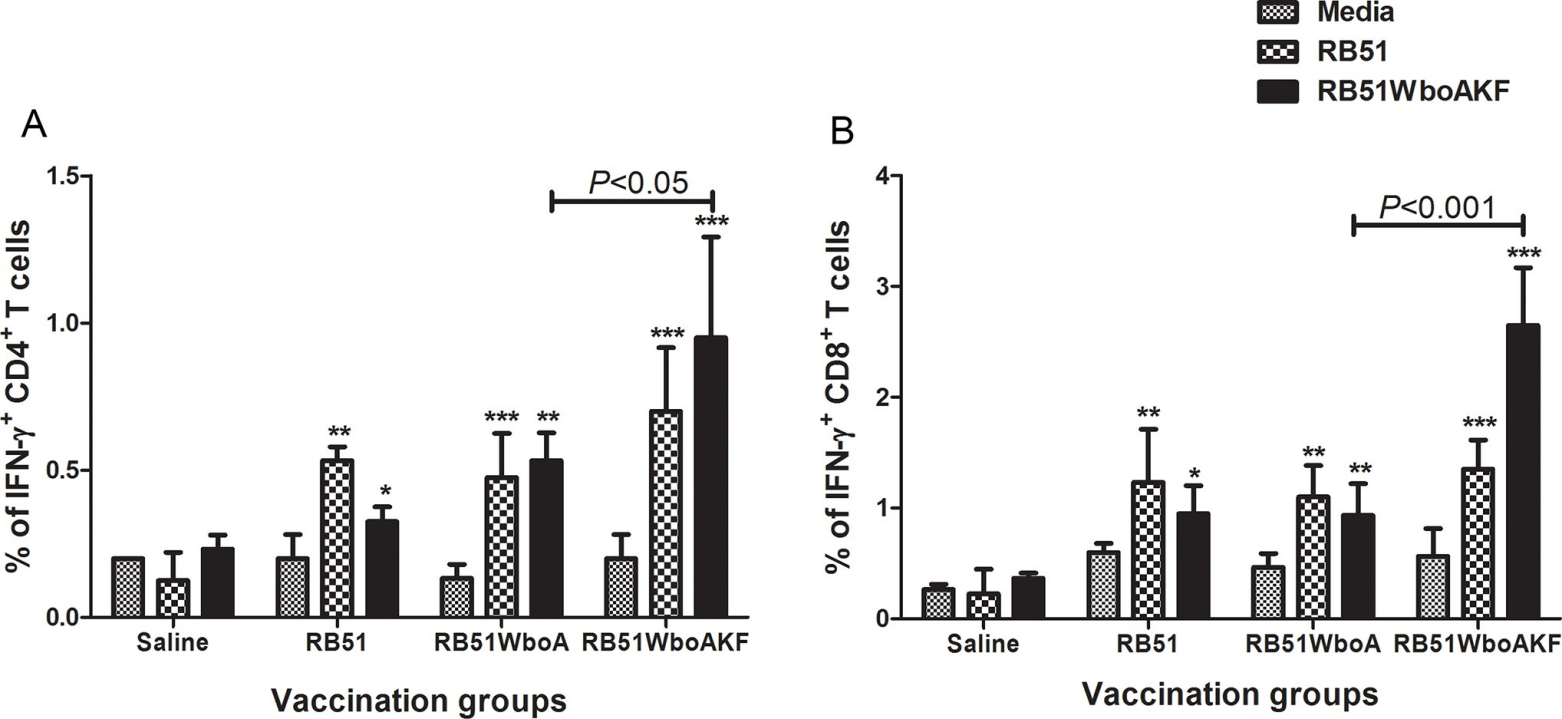
**Flow cytometric analysis showing the percentage of interferon-γ secreting (A) CD4**^**+**^ **and (B) CD8**^**+**^ **T cells in the spleens of immunized mice.** Mice were immunized intra-peritoneally with 10^8^ CFU of RB51, RB51WboA, RB51WboAKF, or inoculated with saline. Their splenocytes were harvested and stimulated *in vitro* with media (unstimulated), heat-killed RB51 or RB51WboAKF. Values are shown as mean ± standard deviation (n = 4). Asterisks indicate statistically significant differences from the corresponding unstimulated control. *, *P* < 0.05; **, *P* < 0.01; ***, *P* < 0.001.

Upon *in vitro* stimulation with RB51 and RB51WboAKF, higher proportions of IFN-γ secreting CD4^+^ and CD8^+^ T cells were detected in all the vaccinated groups of mice but not the saline-inoculated group when compared with the corresponding unstimulated controls (Fig 9). When stimulated with RB51WboAKF, significantly higher proportions of IFN-γ secreting CD4^+^ and CD8^+^ T cells were detected in the RB51WboAKF vaccinated mice when compared to other vaccinated groups of mice (Fig 9).

**Fig 10:**
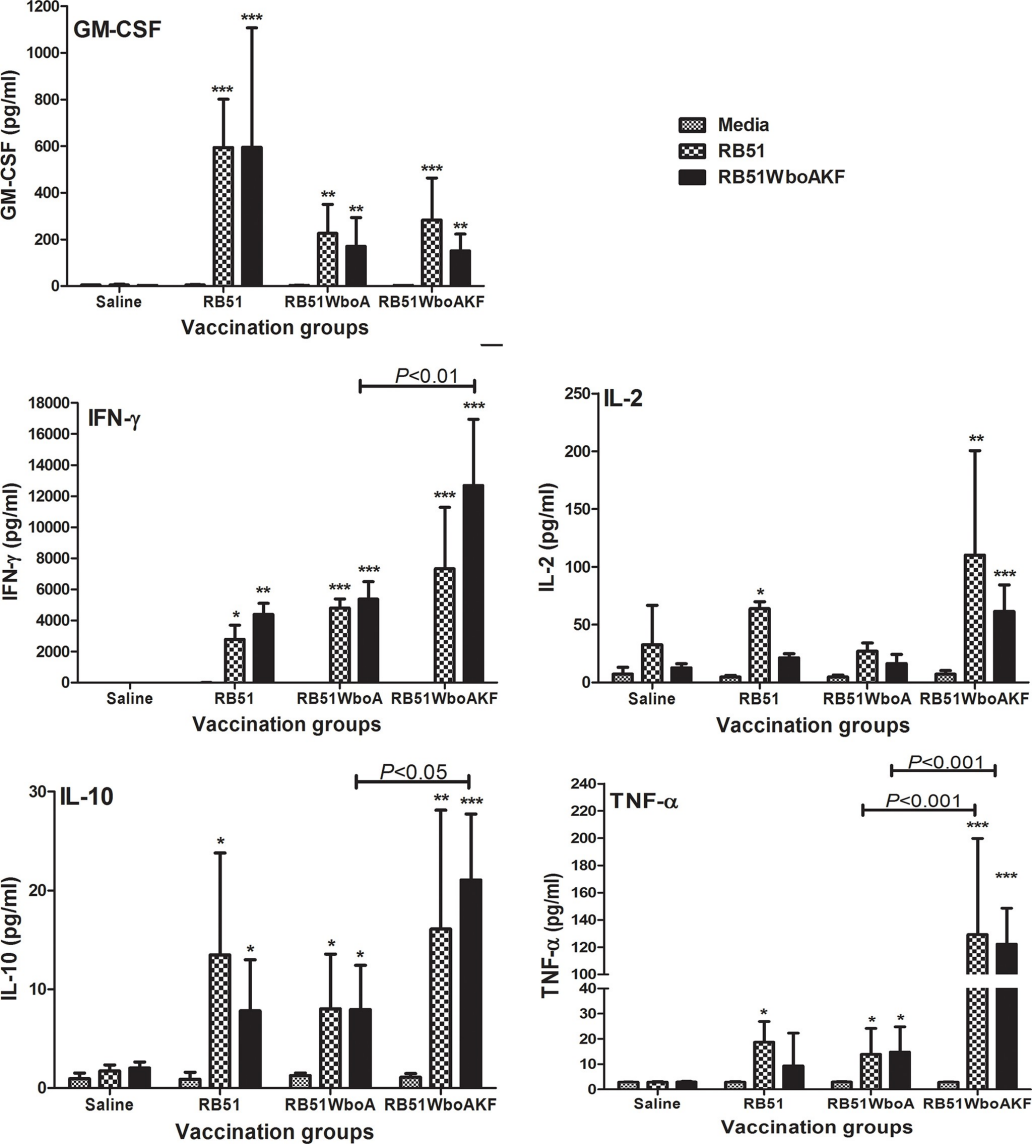
Detection of specific cytokines produced by splenocytes of vaccinated mice. Mice were vaccinated intra-peritoneally with 10^8^ CFU of live RB51, RB51WboA, RB51WboAKF or inoculated with saline. Their splenocytes were harvested and stimulated *in vitro* with media (unstimulated), heat-killed RB51 or RB51WboAKF. Values are shown as mean ± standard deviation (n = 4). Asterisks indicate statistically significant differences from the corresponding unstimulated control. *, *P* < 0.05; **, *P* < 0.01; ***, *P* < 0.001.

Splenocytes of all groups of vaccinated mice secreted significantly increased levels of IFN-γ, GM-CSF and IL-10 upon *in vitro* stimulation with RB51 and RB51WboAKF (Fig 10). The levels of IL-4 and IL-5 in the culture supernatants of all groups of mice were below the detection limit. Stimulation with RB51WboAKF resulted in the secretion of significantly higher levels of IFN-γ and IL-10 by the splenocytes of RB51WboAKF vaccinated group of mice than the RB51WboA vaccinated mice group (Fig 10). Also, upon *in vitro* stimulation with RB51 and RB51WboAKF, significantly higher levels of TNF-α were detected in the culture supernatants of mice vaccinated with RB51WboA and RB51WboAKF, but not the saline-inoculated group, when compared to their unstimulated controls; the level of TNF-α was significantly higher in the RB51WboAKF vaccinated mice group when compared with other vaccinated groups of mice (Fig 10). Higher levels of IL-2 were also detected in the culture supernatants of mice vaccinated with RB51WboAKF upon *in vitro* stimulation with RB51WboAKF (Fig 10).

### 3.7. Enhanced protection against virulent *Brucella* challenge

**Fig 11:**
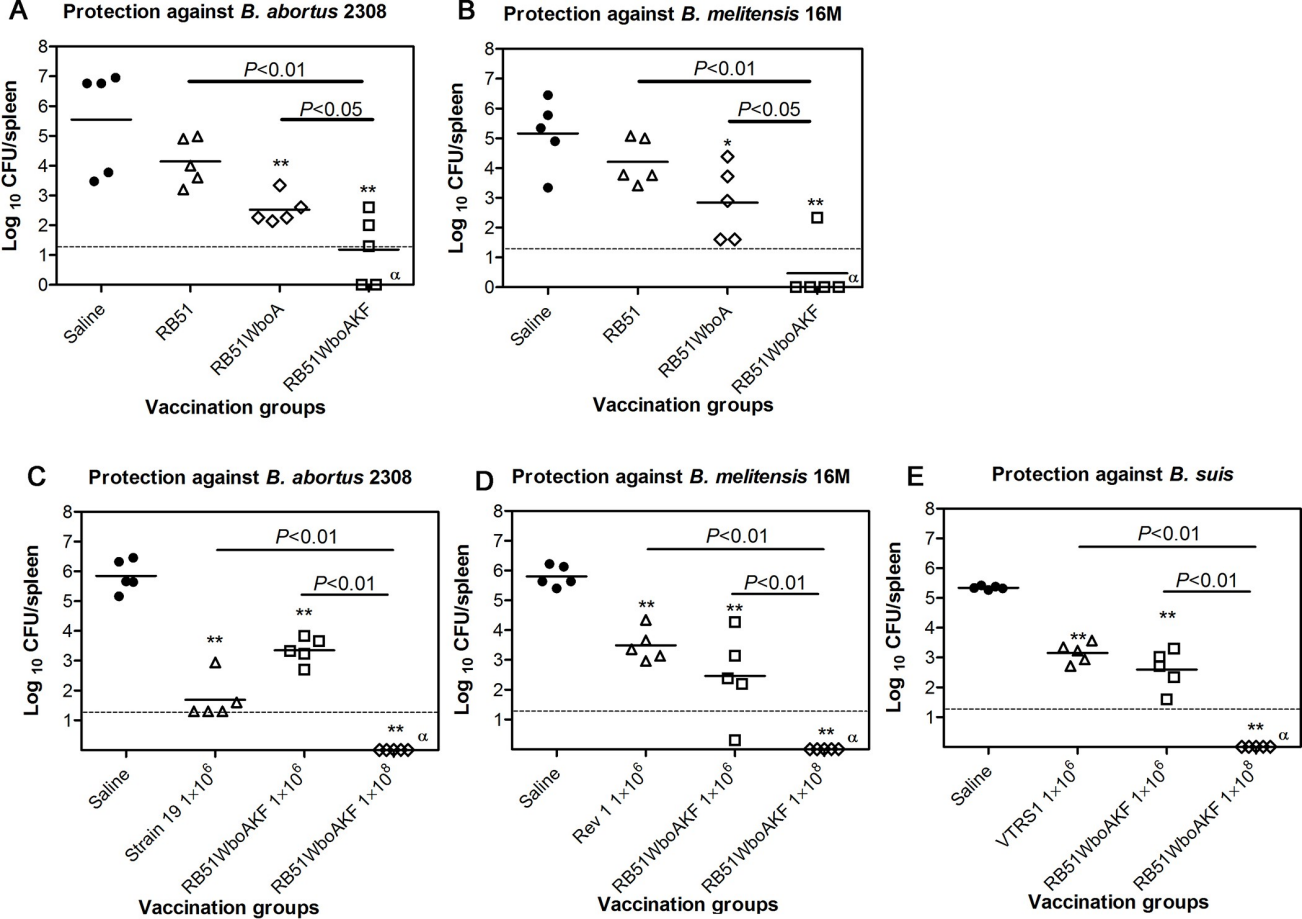
Protection of mice against challenge with virulent strains *B*. *abortus* 2308, *B*. *melitensis* 16M or *B*. *suis* 1330. Mice were vaccinated with the indicated strains or inoculated with saline alone were challenged with (A and C) *B*. *abortus* 2308, (B and D) *B*. *melitensis* 16M and (E) *B*. *suis* 1330, and 2 weeks post-challenge, the mice were euthanized and the *Brucella* CFU in their spleens were determined. Results are shown as mean ± standard deviation (n = 5) of the log CFU of *Brucella* recovered from spleens. Horizontal broken line above the x-axis indicates the lower limit of detection (<20 CFU/spleen). Asterisks indicate statistically significant differences from the corresponding saline group. *, *P* < 0.05; **, *P* < 0.01; ***, *P* < 0.001. α: no bacteria were recovered on plating the entire homogenate.

Vaccination with RB51WboA and RB51WboAKF resulted in significant reduction of splenic bacterial load following challenge with virulent *B*. *abortus* 2308 and *B*. *melitensis* 16M when compared with the saline inoculated group of mice (2.3–4.7 logs protection) (Fig 11A and 11B). Following challenge with virulent *B*. *abortus* 2308 and *B*. *melitensis* 16M, RB51WboAKF immunized group of mice showed the highest reduction in the splenic bacterial load when compared with RB51 and RB51WboA vaccinated groups (1.3–3.7 logs more protection) (Fig 11A and 11B).

Separate protection experiments were carried out to compare the protective efficacy of two different doses of strain RB51WboAKF with that of vaccine strains *B*.*abortus* S19, *B*. *melitensis* Rev 1 or *B*. *suis* VTRS1. At 1× 10^6^ CFU vaccine dose, strain RB51WboAKF induced similar level of protection as that of strain 19, Rev 1, and VTRS1, against challenge with *B*. *abortus* 2308, *B*. *melitensis* 16M, and *B*. *suis* 1330, respectively (2.2–4.2 logs protection) (Fig 11C, 11D and 11E). However, at a higher dose of 1× 10^8^ CFU, strain RB51WboAKF showed the highest protective efficacy against all three virulent *Brucella spp*. challenge and no challenge bacteria were detected in the mice spleens (5.3–5.9 logs protection) (Fig 11C, 11D and 11E).

## 4. Discussion

Our study demonstrated that overexpression of *wbkF* in strain RB51WboA led to higher levels of expression of O-PS. The expressed O-PS was present both on the cell surface and in the cell membrane of strain RB51WboAKF. However, RB51WboAKF still exhibited rough phenotypic characteristics as evidenced by auto-agglutination reaction with acriflavin. This type of simultaneous display of smooth and rough phenotypic characteristics was described previously in a laboratory derived *B*. *melitensis* strain B18 and in a natural isolate of *B*. *melitensis* (strain EP) [19, 28]. The phenotypic dichotomy of strain EP was attributed to the presence of fewer O-PS chains and higher amounts of rough-LPS when compared to the strain 16M [28]. In case of RB51WboAKF strain, the reduced reactivity of whole cells with smooth LPS-specific antibody and the enhanced sensitivity to Polymyxin B when compared to smooth *B*. *neotomae* suggest the presence of fewer surface-exposed O-PS chains and incomplete smooth LPS. However, further studies are needed to confirm this observation.

The O-PS is a major virulence factor of *Brucella* [1, 8, 17]. It inhibits complement-mediated lysis and also impairs phagosome-lysosome fusion, at least during the first few hours after infection [1, 29]. The O-PS of *Brucella* is a homopolymer of N-formylperosamine where the subunits are α-1,2 linked in A-dominant strains, with every fifth residue linked in α -1,3 in M-dominant *Brucella* smooth strains [30]. The complete biosynthetic pathway of O-PS in *Brucella* is yet to be determined; however, studies of homologous protein functions as well as genetic mutations that yield rough *Brucella* have contributed in the prediction of a reliable model pathway [2, 17, 31, 32, 33, 34]. Genome analysis of *Brucella* species has revealed the presence of Wzm and Wzt, which comprise the ABC-transporter system; accordingly, the homopolymeric *Brucella* O-PS is thought to be synthesized by the Wzy-independent pathway [31, 32, 33, 34, 35]. In this pathway, the O-PS biosynthesis is initiated by the transfer of a sugar phosphate onto the isoprenoid carrier lipid, the undecaprenol phosphate or bactoprenol phosphate, leading to the synthesis of the first pyrophosphoryl-bactoprenol-linked oligosaccharide intermediate [34, 35]. Subsequently, specific glycosyl transferases catalyze the transfer of successive monosaccharide residues from NDP-activated sugar precursors to the non-reducing end of the growing polysaccharide chain [34, 35]. The completed O-PS is translocated across the inner membrane to the periplasmic face of the bacteria by an ATP-driven transporter system. In the periplasm, the O-antigen ligase attaches the O-PS to the lipid A-core molecule, following which the full length smooth LPS is transported to the outer membrane by a trans-envelope protein machinery [36].

In the predicted model of *Brucella* O-PS synthesis, WbkF and WbkD catalyze sequential steps that lead to priming of the bactoprenol phosphate [31]. Disruption of *wbkD* in *B*. *melitensis* leads to rough LPS with a complete core structure [31, 37]. Based on amino acid sequence homology, it is predicted that *wbkD* encodes an epimerase/dehydratase that takes part in the synthesis of 2-N-acetamido-2,6-dideoxyglucose (N-acetyl-quinovosamine) [31, 38] that has been found in the smooth LPS [3, 7, 39]. It is proposed that N-acetyl-quinovosamine acts as a primer sugar substrate for WbkF (polyisoprenyl-phosphate N-acetylhexosamine-1-phosphate transferase) for the synthesis of bactoprenol-primed oligosaccharide intermediate [31, 37]. However, if N-acetyl-quinovosamine is the undecaprenol-priming residue and the first sugar of *Brucella* O-PS chain remains to be confirmed.

Subsequently, glycosyltransferases, including WboA, polymerize N-formylperosamine onto the bactoprenol-primed N-acetylaminosugar [31]. Previous studies have demonstrated that mutations in *wbkF*, as well as *wbkD* and *wboA*, in smooth *Brucella* species yield rough strains that cannot produce O-PS [31, 32, 33]. Our findings showed that overexpression of *wbkD*, unlike *wbkF*, in strain RB51WboA did not change the level of O-PS expression. This suggests that increasing the levels of bactoprenol-primed precursors, but not N-acetylquinovosamine itself, in strain RB51WboA further enhances the expression of O-PS.

In spite of expression of higher O-PS levels, the attenuation characteristic of strain RB51WboAKF did not differ from the parent strain RB51. Based on the increased resistance to Polymyxin B treatment, we had expected that strain RB51WboAKF would persist longer in mice organs than strain RB51. The lack of any increase in the virulence characteristic of strain RB51WboAKF suggests that the attenuation of strain RB51 is because of mutations in multiple yet to be identified genes. A limitation of this study, however, was that we used only 3 mice per group; increasing the number of animals per group may be necessary to detect smaller, yet statistically significant differences, between the two groups.

Th1-biased immunity is central to protection against brucellosis [10, 11, 12, 31, 40]. Vaccination of mice with strain RB51WboAKF resulted in the induction of Th1 type of immunity, as demonstrated by the production of higher levels of IgG2a and IgG3 subisotype of LPS-specific antibodies and induction of antigen-specific CD4^+^ and CD8^+^ T cells that secrete IFN-γ. The role of IFN-γ producing T cells in anti-*Brucella* immunity is well documented [10, 12, 16, 26, 31, 33, 40]. The vaccine-induced T cells were also capable of secreting significantly increased levels of TNF-α. Previous reports have demonstrated the role of TNF-α in host resistance against intracellular microorganisms [41, 42, 43]. The exact mechanism by which TNF-α may contribute to enhanced immunity against *Brucella* is still a subject of some debate. TNF-α plays a crucial role in enhancing IL-12 production which in turn upregulates the secretion of IFN-γ by T cells and natural killer (NK) cells [44]. Depletion of IL-12 leads to a decrease in the production of IFN-γ and exacerbates pathology in many intracellular infections including *Brucella* infection [44, 45, 46]. Our study demonstrated the concurrent increase in IL-12 and IFN-γ in the serum of RB51WboAKF vaccinated mice in as early as day 1 after inoculation. Similar to previous reports, the production of IL-12 was transitory and ceased even while bacteria still persisted in significant numbers in mice [47]. This regulation of IL-12 is necessary to prevent tissue damage and is said to be mediated by IL-10 [44, 47]. While IL-12 mediates antibacterial resistance by regulating levels of IFN-γ, the effect of TNF-α is primarily IFN-γ-independent [47]. Even in the absence of IFN-γ, TNF-α enhances *Brucella* killing by activating macrophages [48].

RB51WboAKF vaccine conferred significantly higher protection against virulent *Brucella* species than strains RB51, S19, Rev1 and VTRS1. When similar vaccine doses were compared, RB51WboAKF vaccine at a dose of 1× 10^6^ CFU conferred similar level of immunity as that of strains S19, Rev1 and VTRS1 against *B*. *abortus*, *B*. *melitensis* and *B*. *suis* challenge infection, respectively. In this study, we did not assess the immune responses induced by RB51WboAKF at the lower dose. Both T cell-mediated responses as well as smooth LPS-specific antibodies play a role in providing enhanced protection against brucellosis in murine models [11, 31, 49, 50, 51]. Further studies are needed to determine the effect of different vaccine doses on RB51WboAKF-induced immune responses and protective efficacy against homologous (i.e., *B*. *abortus*) and heterologous (i.e., *B*. *melitensis* and *B*. *suis*) challenge. Also, studies in natural hosts are necessary to verify if the observed gains in the protective efficacy by RB51WboAKF in murine models is translatable to application in the targeted species.

In conclusion, our results suggest that expression of increased levels of O-PS in RB51 does not alter its rough phenotype and attenuation characteristics. However, such a modified RB51 strain (i.e., RB51WboAKF) is more immunogenic as a vaccine, capable of conferring enhanced protection against infection with virulent *Brucella spp* in the mouse model.

## Supporting information

S1 Fig Detection of RB51-specific IgM, IgG, IgG1, IgG2a, IgG2b and IgG3 antibodies in serum of mice inoculated with RB51, RB51WboA, RB51WboAKF or saline. Serum samples were collected at 3 and 6 weeks after vaccination, were diluted 1 in 200 and assayed for the presence of RB51-specific antibodies by indirect ELISA. Results are shown as mean ± standard deviation (n = 4) of absorbance of the color developed. Asterisks indicate statistically significant differences from the corresponding saline group. *, *P* < 0.05; **, *P* < 0.01; ***, *P* < 0.001. OD, optical density.
(TIF)
