# Supplementary figures and images for: Overexpression of wbkF gene in Brucella abortus RB51WboA leads to increased O-polysaccharide expression and enhanced vaccine efficacy against B. abortus 2308, B. melitensis 16M, and B. suis 1330 in a murine brucellosis model
Source: PLoS One. 2019 Mar 11;14(3):e0213587. doi: 10.1371/journal.pone.0213587 (PMC6411116; doi:10.1371/journal.pone.0213587)

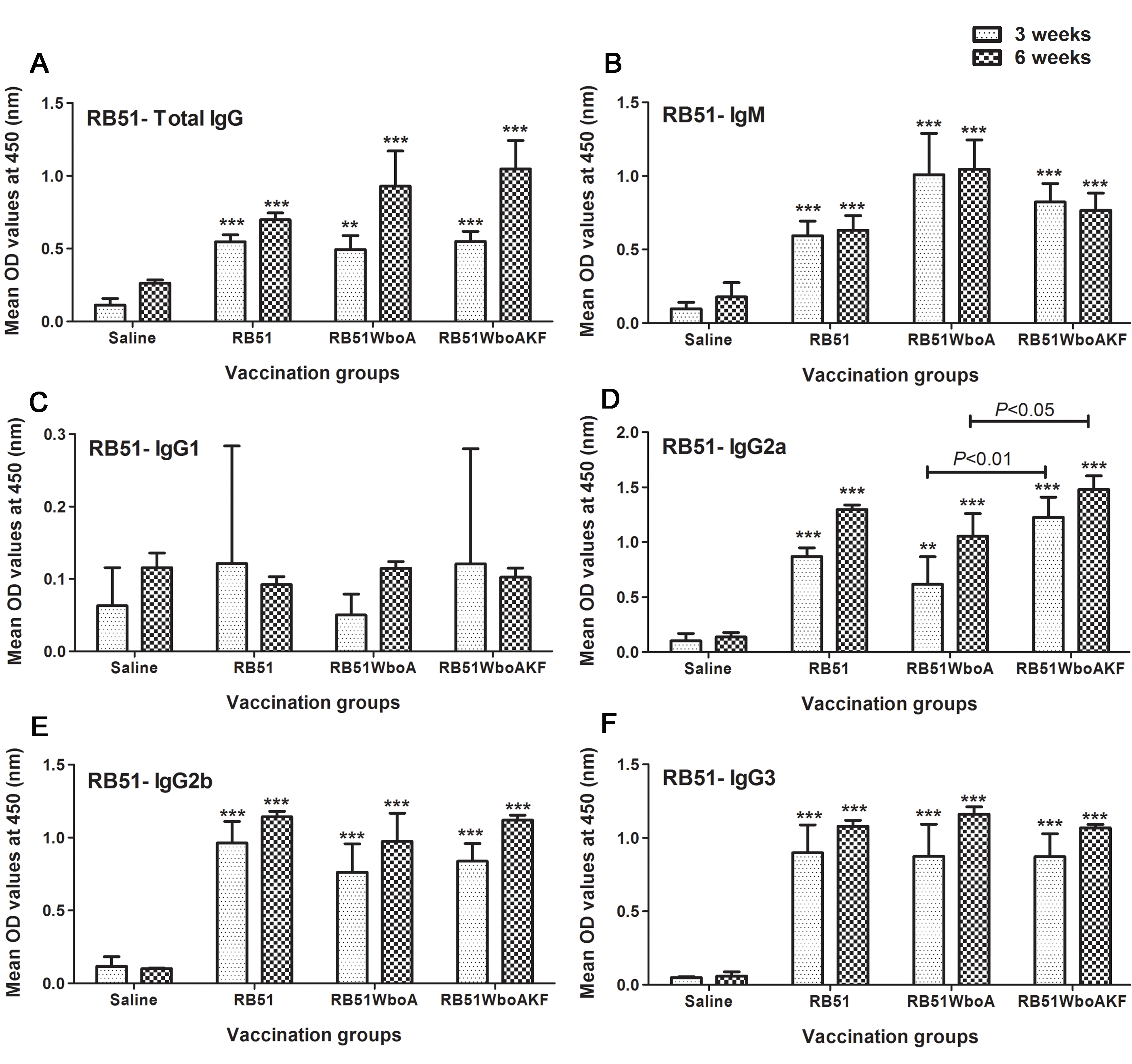

Supplement: S1 Fig — Serum samples were collected at 3 and 6 weeks after vaccination, were diluted 1 in 200 and assayed for the presence of RB51-specific antibodies by indirect ELISA. Results are shown as mean ± standard deviation (n = 4) of absorbance of the color developed. Asterisks indicate statistically significant differences from the corresponding saline group. *, P < 0.05; **, P < 0.01; ***, P < 0.001. OD, optical density. (TIF) [file pone.0213587.s001.tif]
